# The Neural Correlates of Consciousness: A Spectral Exponent Approach to Diagnosing Disorders of Consciousness

**DOI:** 10.3390/brainsci15040377

**Published:** 2025-04-04

**Authors:** Ying Zhao, Anqi Wang, Weiqiao Zhao, Nantu Hu, Steven Laureys, Haibo Di

**Affiliations:** 1 International Unresponsive Wakefulness Syndrome and Consciousness Science Institute, Hangzhou Normal University, Hangzhou 311121, China; 2 Department of Basic Medicine, Hangzhou Normal University, Hangzhou 311121, China; 3 Coma Science Group, GIGA-Consciousness, University of Liège, 4000 Liège, Belgium

**Keywords:** disorder of consciousness, spectral exponent, EEG biomarker

## Abstract

Background/Objectives: Disorder of consciousness (DoC) poses diagnostic challenges due to behavioral assessment limitations. This study evaluates the spectral exponent (SE)—a neurophysiological biomarker quantifying the decay slope of electroencephalography (EEG) aperiodic activity—as an objective tool for consciousness stratification and clinical behavior scores correlation. Methods: The study involved 15 DoC patients, nine conscious brain-injured controls (BI), and 23 healthy controls (HC). Resting-state 32-channel EEG data were analyzed to compute SE across broadband (1–40 Hz) and narrowband (1–20 Hz, 20–40 Hz). Statistical frameworks included Bonferroni-corrected Kruskal–Wallis H tests, Bayesian ANOVA, and correlation analyses with CRS-R behavioral scores. Results: Narrowband SE (1–20 Hz) showed superior diagnostic sensitivity, differentiating DoC from controls (HC vs. DoC: *p* < 0.0001; BI vs. DoC: *p* = 0.0006) and MCS from VS/UWS (*p* = 0.0014). SE correlated positively with CRS-R index (1–20 Hz: r = 0.590, *p* = 0.021) and visual subscale (1–20 Hz: r = 0.684, *p* = 0.005). High-frequency (20–40 Hz) SE exhibited inconsistent results. Longitudinal tracking in an individual revealed a reduction in SE negativity, a flattening of the 1/f slope, and behavioral recovery occurring in parallel. Conclusions: Narrowband SE (1–20 Hz) is a robust biomarker for consciousness quantification, overcoming behavioral assessment subjectivity. Its correlation with visual function highlights potential clinical utility. Future studies should validate SE in larger cohorts and integrate multimodal neuroimaging.

## 1. Introduction

Consciousness, phenomenologically defined as the subjective integration of self-awareness and environmental perception, arises from the spatiotemporal coordination of thalamocortical systems and distributed large-scale cortical networks [1,2]. Modern neuroscientific frameworks operationalize this phenomenon through two neurobiologically distinct yet synergistic components. The first, arousal (or vigilance), constitutes the neurophysiological foundation of wakefulness, mediated by ascending reticular activating system (ARAS) projections that gate thalamocortical excitability. The second, awareness (or content-specific consciousness), emerges from the global integration of multimodal information across frontoparietal association cortices, enabling hierarchical perceptual binding [3,4]. This dual-component architecture not only aligns with clinical dissociations observed in consciousness disorders but also converges with neurocomputational models emphasizing integrated information capacity as a consciousness metric [2,4].

Disorders of consciousness (DoC), characterized by persistent impairment of environmental awareness and self-representation post-cerebral injury, pathologically manifest as disrupted thalamocortical connectivity or cortico–ocortical disintegration [4]. Clinically, the Coma Recovery Scale-Revised (CRS-R) remains the diagnostic gold standard, categorizing patients into coma, vegetative state/unresponsive wakefulness syndrome (VS/UWS), minimally conscious state (MCS), and emergence from MCS (EMCS) through standardized behavioral protocols [5,6].

Coma manifests clinically as persistent eye closure, absence of sleep–wake cycles, and unresponsiveness to spontaneous or external stimuli [7,8]. Typically observed in the acute phase (days to weeks) post-brain injury, it may progress to brain death or transition to partial consciousness recovery. Patients in VS/UWS regain sleep–wake cycles but retain only non-volitional reflexive behaviors. Consciousness levels may progressively recover to MCS, where patients demonstrate intermittent and reproducible behavioral responses to simple commands, along with evidence of language comprehension, visual tracking, and localized pain responses [8,9]. The transition to EMCS occurs when patients regain functional object use or autonomous communication capacity, marking substantial improvement in consciousness levels. Notably, the differential diagnosis between VS/UWS and MCS relies solely on behavioral indicators of consciousness, lacking systematic investigation of covert awareness. Substantial clinical evidence reveals that a subset of behaviorally unresponsive patients demonstrates modulations in cerebral metabolic activity during specific task paradigms. These patients exhibiting dissociation between behavioral and neuroimaging evidence are operationally defined as MCS* individuals [10].

However, CRS-R’s diagnostic utility is fundamentally limited by three confounders: (1) vulnerability to transient arousal fluctuations; (2) reliance on intact motor pathways for response generation; and (3) inter-rater variability in detecting subtle signs of awareness. These limitations collectively result in misclassification rates of 30–40% [8,9], underscoring the urgent need for objective biomarkers that dissociate arousal-awareness dynamics and circumvent motor-dependent behavioral proxies.

The limitations of behavioral scales have driven the evolution of multimodal assessment frameworks that integrate neuroimaging and electrophysiological techniques to improve the detection of covert awareness [11,12]. Commonly employed modalities include fluorodeoxyglucose-positron emission tomography (FDG-PET) and functional magnetic resonance imaging (fMRI), which decode consciousness through metabolic patterns and functional activation profiles, respectively. While these methods provide complementary insights into large-scale network dynamics and causal interactions, their clinical translation is constrained by cost, accessibility, or invasiveness. Based on the Perturbational Complexity Index (PCI) proposed by Integrated Information Theory (IIT) [13], cortical information was quantified through Transcranial Magnetic Stimulation combined with EEG (TMS-EEG) integration ability, using 0.31 as the threshold for the presence or absence of consciousness. However, TMS-EEG requires an electromagnetically shielded environment and lacks a standardized stimulation protocol, limiting its bedside application [14]. Resting-state EEG, despite its limited spatial resolution and susceptibility to motion artifacts, serves as a pragmatic bedside tool for consciousness monitoring due to its portability, cost-effectiveness, and millisecond-scale temporal resolution, thereby addressing the practical demands of DoC diagnosis.

The ABCD neurophysiological typology, grounded in corticothalamic deafferentation dynamics, categorizes human EEG into four distinct spectral patterns reflecting hierarchical recovery of thalamocortical integration [15]. Type A, dominated by delta activity (<4 Hz), signify complete functional corticothalamic deafferentation, analogous to experimental models of thalamic input deprivation. Type B, characterized by theta oscillations (5–7 Hz), emerge from sparse afferent drive to neocortical pyramidal neurons, with coherence peaks at 7 Hz suggesting diffusely coupled intrinsic oscillations. Type C, marked by theta-beta comodulation (15–40 Hz), reflect thalamo-cortical dysrhythmia, where burst-mode thalamic firing interacts with partially preserved cortical networks. Type D, exhibiting physiological alpha (8–12 Hz) and beta rhythms, indicate restored thalamic tonic firing and normal cortical integration.

Recent advancements in neuroimaging have unveiled characteristic electrophysiological signatures of consciousness loss. Beyond classical EEG slowing phenomena (elevated delta power with alpha peak anteriorization) [16,17], the spectral decay slope of aperiodic oscillations (1/f characteristics), quantified as the spectral exponent (SE), demonstrates robust correlations with consciousness states. SE captures spatiotemporal dynamics of neural noise by measuring the attenuation rate of power spectral density (PSD) in log–log coordinates [18]. Empirical validations span diverse experimental paradigms: anesthesia-induced unresponsiveness [19], physiological sleep [20], and acute cerebral injury models [21] all demonstrate that diminished consciousness levels correlate with steepened 1/f slopes (more negative SE values). This phenomenon likely reflects a shift in excitation–inhibition (E-I) balance toward inhibitory dominance. Computational models indicate that heightened inhibitory synaptic activity suppresses high-frequency oscillations, thereby accelerating PSD decay [22,23], a mechanism corroborated by primate studies showing subcortical PSD steepening during propofol-induced unconsciousness [22].

Crucially, SE’s translational potential hinges on frequency band optimization. Two key challenges emerge: First, residual thalamic bursting activity in Type C DoC patients introduces high-frequency (>20 Hz) spectral peaks that distort broadband (1–40 Hz) SE estimation, as per the ABCD EEG typology [13]. Second, dendritic low-pass filtering imposes a characteristic spectral “knee point” (~20 Hz) in neural population dynamics, constraining signal transmission fidelity beyond this threshold [24]. These observations collectively advocate for narrowband (1–20 Hz) analysis to isolate consciousness-specific neural dynamics. However, despite these mechanistic insights, critical gaps persist in SE’s clinical validation for chronic DoC populations. Specifically, three unresolved issues demand systematic investigation: (1) frequency-dependent classification sensitivity across broadband vs. narrowband ranges; (2) behavioral correlates with standardized clinical scales (e.g., CRS-R); and (3) robustness to confounding effects of structural brain injury.

To address these knowledge gaps, this study pursues three pivotal objectives: (1) frequency-specific diagnostic sensitivity—comparative evaluation of SE’s classification performance across broadband (1–40 Hz) versus narrowband (1–20 Hz, 20–40 Hz) ranges; (2) Behavioral correlation mapping—quantitative assessment of SE’s association with CRS-R composite scores and modality-specific subscales (particularly visual function); (3) Structural lesion robustness—determination of SE’s biological specificity to functional thalamocortical dynamics versus structural damage confounds. We hypothesize that narrowband SE (1–20 Hz) will outperform broadband metrics in discriminating VS/UWS from MCS, independent of structural lesion burden, by selectively indexing thalamocortical circuit integrity essential for conscious processing.

## 2. Materials and Methods

### 2.1. Participant Recruitment

The study cohort involved 17 patients with prolonged disorders of consciousness (pDoC), 10 conscious brain-injured controls (BI), and 23 healthy controls (HC) recruited from Shanghai Yongci Rehabilitation Hospital between October 2023 and April 2024. Written informed consent was obtained from all participants or their legal guardians if patients lacked decision-making capacity. The study protocol was approved by the Ethics Committee of Hangzhou Normal University (Approval No: 20230630).

Inclusion Criteria for DoC Patients: (1) Diagnosed as VS/UWS, MCS, or EMCS via repeated CRS-R assessments within 1 week; (2) Aged 16–80 years; (3) Stable vital signs; (4) Absence of large skull defects or scalp deformities; (5) Time since injury duration >28 days; (6) No sedative use within 12 h prior to EEG recording. Exclusion Criteria: (1) Malignant space-occupying lesions (e.g., carcinomas, sarcomas); (2) Frequent involuntary limb movements or facial spasms; (3) Skin lesions or scabs at electrode sites; (4) Persistent eye closure (>48 h) unresponsive to arousal protocols.

This study screened 36 patients with DoC, excluding 19 cases due to the following reasons: inability to maintain an alert state (n = 2), presence of cranial drainage tubes (n = 1), pulmonary malignant space-occupying lesions (n = 1), unhealed cerebral wounds (n = 4), fever and convulsive conditions (n = 3), hemodynamically unstable vital signs (n = 3), and family-initiated withdrawal prior to data collection (n = 5). Of these, two patients did not fulfill exclusion criterion 2 and three patients did not fulfill exclusion criterion 4.

HC: Age-matched HCs were recruited from patients’ families, meeting criteria 2, 4, and 6 of the DoC inclusion criteria.

BI Controls: The BI control group comprised patients with structural brain injuries (e.g., ischemic stroke, cerebral contusion) and preserved consciousness, with exclusion criteria identical to those of the DoC group except for the level of consciousness.

### 2.2. Clinical Assessment

Each DoC patient underwent daily CRS-R evaluations. To minimize bias, EEG operators were blinded to behavioral assessments. Consciousness levels were determined by the highest CRS-R score across five repeated evaluations within 1 week [25]. The CRS-R, a validated scale for assessing residual consciousness in severe brain injury, comprises six subscales (auditory, motor, visual, oromotor/verbal, communication, and arousal) with hierarchically organized items to detect subtle signs of awareness.

### 2.3. CRS-R Index Calculation

The CRS-R index (range: 0–100) was computed using a validated logistic model, with a cutoff of 8.315 differentiating VS/UWS from MCS [26]. Reflexive (subcortical) and voluntary (cortical) behaviors were weighted across subscales, integrated via a transformation matrix, and adjusted for arousal levels.

### 2.4. EEG Acquisition

The EEG (32-channel, 1000 Hz sampling, electrode impedance <10 kΩ) captured eyes-open resting-state activity over 8 min according to the international 10–20 system. FCz and AFz served as reference and ground, respectively. Real-time video monitoring (25 fps) enabled artifact annotation, while arousal protocols (tactile stimulation/verbal prompts) maintained vigilance. Sleepiness (prolonged eye closure >10 s) was counteracted using CRS-R arousal protocols (deep pressure stimulation) for DoC patients or verbal prompts for others. Data were exported in EDF format.

One BI participant was excluded from analysis due to excessive voluntary limb movements and unwillingness to comply with instructions during data acquisition, resulting in severe electromyographic (EMG) artifacts. Two pDoC patients were excluded because of their inability to maintain eye-opening/sustained wakefulness during EEG recording.

### 2.5. EEG Preprocessing

Data were processed offline using EEGLAB 2021 (MATLAB R2018a) with the following steps: (1) Artifact rejection: Video-identified motion artifacts were removed; (2) Filtering: 5th-order Butterworth IIR bandpass (0.5–50 Hz) and 50 Hz notch filtering; (3) Channel interpolation: Bad channels reconstructed via spline interpolation; (4) ICA denoising: Ocular and muscular artifacts removed; (5) Epoch segmentation: Clean data segmented into non-overlapping 10 s epochs with average referencing. EEG segments with amplitudes exceeding ±100 μV were removed. For each participant, 30 artifact-free EEG epochs were selected for subsequent analysis.

### 2.6. SE Estimation

PSD was calculated using Welch’s method (2 s Hanning window, 50% overlap). SE was estimated via linear regression on log-log transformed PSD within three frequency bands (1–20 Hz, 20–40 Hz, and 1–40 Hz), reflecting the decay rate of EEG slowing.

### 2.7. Statistical Analysis

The SE values at the whole-brain level were computed by first averaging across all channels within each EEG epoch and subsequently averaging across all epochs. Group differences were assessed using Bonferroni-corrected Kruskal–Wallis H tests and unpaired *t*-tests. Pearson/Spearman correlations evaluated associations between SE and CRS-R indices/subscales. Analyses were performed in SPSS 26.0 and R 4.4.2 (α = 0.05, two-tailed). We also employed a Bayesian statistical framework to address small-sample limitations. A Bayesian one-way ANOVA with Jeffreys–Zellner–Siow priors was used for their robustness to variance heterogeneity in neurological cohorts, calculating Bayes factors (BF10) via the R BayesFactor package (Kass and Raftery’s criteria: BF10 > 3 indicates moderate evidence, >10 strong evidence, >30 very strong evidence.). Sensitivity analyses with alternative priors confirmed result stability. Moreover, this approach addresses the limitation of traditional frequentist methods in directly quantifying evidence for the null hypothesis (H0), as the Bayesian factor BF01-defined reciprocally as 1/BF10-provides a statistical metric to rigorously quantify evidence of equivalence or absence of difference. Significant effects underwent Bayesian independent t-tests with Bonferroni correction, cross-validated against frequentist ANOVA. For DoC subgroups, Bayesian t-tests modeled effect-size distributions (δ~Cauchy (0, 0.707)), circumventing normality/homogeneity assumptions through posterior credible intervals and BF10, establishing a tripartite “statistical significance-evidence strength-effect magnitude” framework for heterogeneous brain injury cohorts.

### 2.8. Follow-Up Assessments

For enrolled DoC patients remaining hospitalized, follow-up evaluations were conducted three months post-enrollment by the original consciousness assessment operator. Five CRS-R assessments were administered within one week to minimize diurnal fluctuation confounds. Patients demonstrating significant improvements in clinical manifestations or CRS-R scores underwent individualized longitudinal case analysis. FDG-PET imaging was performed contingent upon obtaining informed consent from legal guardians.

## 3. Results

### 3.1. Demographic Characteristics

The final cohort comprised 15 DoC patients (mean age: 53.3 ± 15.4 years; three females; mean post-injury duration: 221.53 ± 161.31 days; six traumatic brain injury, nine MCS) shown in Table 1, nine conscious brain injury (BI) patients (mean age: 48.1 ± 16.4 years; one female) in Table 2, and 23 healthy controls (HC; mean age: 51.2 ± 12.2 years). Demographic characteristics are summarized in Table 3.

### 3.2. Group-Level Analysis

**Figure 1:**
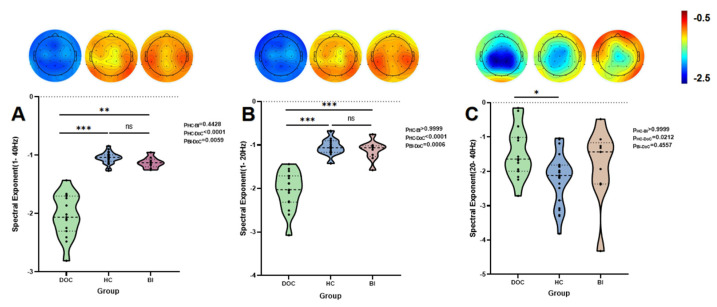
Intergroup classification results of SE. Note: (**A**) Statistical significance and whole-brain spatial distribution of SE in the 1–40 Hz band; (**B**) SE results and topographic patterns in the 1–20 Hz band; (**C**) SE analysis and whole-brain distribution in the 20–40 Hz band. Significance levels: *** *p* < 0.001, ** *p* < 0.01,* *p* < 0.05 (Bonferroni-corrected).

Significant differences in global SE were observed across frequency bands. In the 1–40 Hz range, the HC and BI group exhibited significantly higher SE values compared to the DoC group (HC vs. DoC: *p* < 0.0001; BI vs. DoC: *p* = 0.0059), with no significant difference between HC and BI groups (*p* = 0.4428). Similar patterns emerged in the 1–20 Hz band, where both HC and BI groups demonstrated markedly elevated SE relative to DoC (HC vs. DoC: *p* < 0.0001; BI vs. DoC: *p* = 0.0006), while HC-BI comparisons remained non-significant (*p* > 0.9999). In contrast, the 20–40 Hz band revealed lower SE in DoC compared to HC (*p* = 0.0212), but no significant differences between DoC and BI (*p* = 0.4557) or BI and HC (*p* > 0.9999). Bayesian ANOVA demonstrated decisive group differences in the 1–40 Hz band (BF_10_ = 5.63 × 1013, η^2^ = 0.81), with post hoc analyses confirming significantly higher SE values in HC and BI groups compared to DoC (BF_10_ > 104), while HC-BI comparisons showed no evidence for differences (BF01 > 1). Similar robustness emerged in the 1–20 Hz band (BF_10_ = 5.996 × 109, η^2^ = 0.71), though discriminative capacity diminished in the 20–40 Hz range (BF_10_ = 2.84, η^2^ = 0.15), where pairwise comparisons yielded inconclusive evidence (BF_10_ < 3). These findings validate 1–20 Hz as a reliable biomarker for consciousness impairment, while highlighting limited utility of high-frequency (>20 Hz) spectral features in group stratification. Complete statistical results are summarized in Table 4, with group-level SE distributions visualized in Figure 1.

### 3.3. Results of Subgroup Analysis

**Figure 2:**
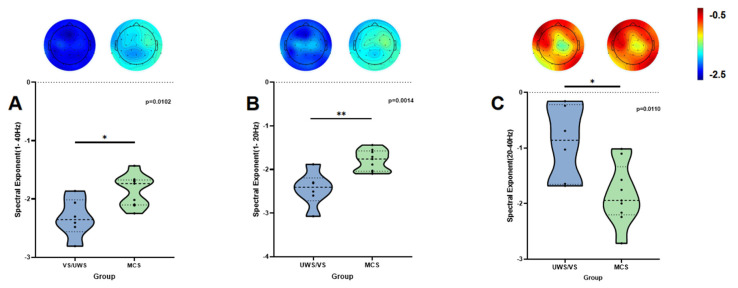
Intragroup classification of DoC results of SE. Note: (**A**) Statistical results and whole-brain distribution of SE within the 1–40 Hz frequency band; (**B**) Statistical results and whole-brain distribution of SE within the 1–20 Hz frequency band; (**C**) Statistical results and whole-brain distribution of SE within the 20–40 Hz frequency band. Significance levels: ** *p* < 0.01,* *p* < 0.05.

Significant statistical differences were observed between the MCS and VS/UWS groups across the following frequency bands: 1–40 Hz (*p* = 0.0102; Figure 2A), 1–20 Hz (*p* = 0.0014; Figure 2B), and 20–40 Hz (*p* = 0.0110; Figure 2C). These findings indicate notable intergroup distinctions in neural oscillatory activity within the specified spectral ranges. Bayesian independent t-tests quantified evidence strength across frequency bands: In the 1–40 Hz range, a moderate evidence level (BF10 = 5.10) supported group differences, while the 1–20 Hz band exhibited decisive evidence (BF10 = 21.61), aligning with highly significant frequentist results (*p* < 0.001). Despite traditional significance in the 20–40 Hz band (*p* = 0.011), Bayesian analysis revealed only moderate support (BF10 = 4.84) alongside a counterintuitive effect direction (Cohen’s d = −1.56), cautioning against overinterpretation of high-frequency biomarkers. Collectively, SE metrics (1–40 Hz, 1–20 Hz) demonstrated robust discriminative capacity for MCS and VS/UWS stratification, whereas conflicting high-frequency (20–40 Hz) outcomes likely reflect frequency-specific neural mechanisms underlying consciousness disorders. Comprehensive numerical values and associated statistical evaluations are summarized in Table 5 and Figure 2.

### 3.4. Correlation Analysis

**Figure 3:**
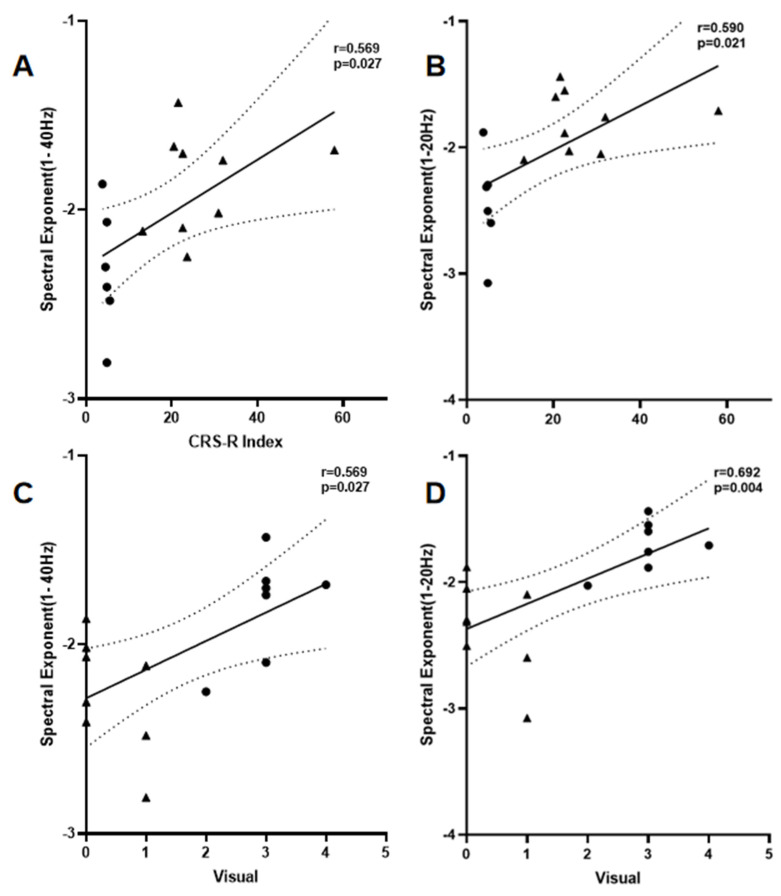
Correlation analysis between SE and behavioral scores. Notes: (**A**) Correlation of 1–40 Hz SE with CRS-R index; (**B**) Correlation of 1–20 Hz SE with CRS-R index; (**C**) Correlation of 1–40 Hz SE with CRS-R visual subscale; (**D**) Correlation of 1–20 Hz SE with CRS-R visual subscale. Dashed lines represent 95% confidence intervals. Circles represent patients in the MCS; triangles represent patients with VS/UWS.

(1) Correlation Between SE and CRS-R Index: The global mean SE value in the 1–40 Hz band demonstrated a significant positive correlation with the Coma Recovery Scale-Revised (CRS-R) index (r = 0.569, *p* = 0.0268, Figure 3A). Similarly, the 1–20 Hz global mean SE value exhibited a significant positive correlation with the CRS-R index (r = 0.590, *p* = 0.0206, Figure 3B). In contrast, the 20–40 Hz band showed a moderately strong negative correlation (r = −0.459); however, this relationship lacked statistical significance (*p* > 0.05) and was excluded from further investigation.

(2) Correlation Between SE and CRS-R Visual Subscale: The visual subscale of the CRS-R displayed significant moderate-to-strong correlations with global SE values in both the 1–40 Hz (r = 0.566, *p* = 0.0278, Figure 3C) and 1–20 Hz (r = 0.684, *p* = 0.0049, Figure 3D) frequency bands. Detailed statistical outcomes are presented in Table 6.

### 3.5. Individual Analysis

**Figure 4:**
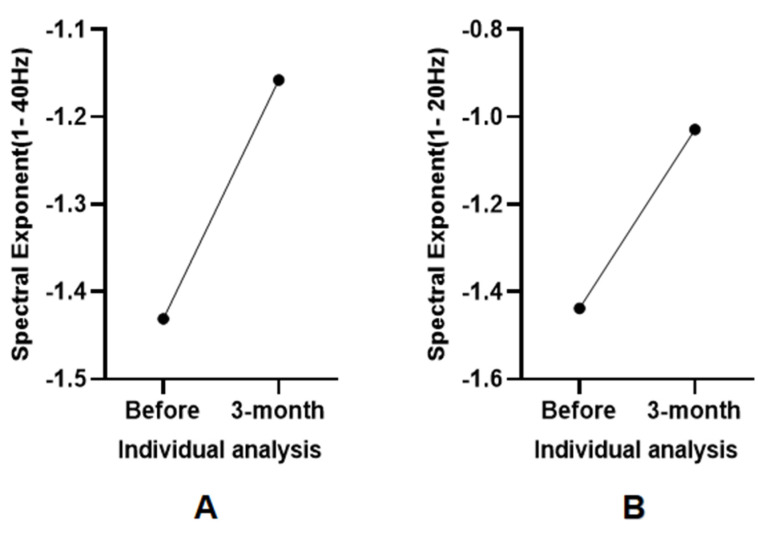
Longitudinal comparison of SE in Patient 5 before and after recovery. Notes: (**A**) SE comparison in the 1–40 Hz band. (**B**) SE comparison in the 1–20 Hz band.

Among the 15 DoC patients analyzed, only Patient 5 regained full consciousness after three months of rehabilitation. At baseline, Patient 5 exhibited a CRS-R score of 1-3-1-1-0-2 (CRS-R index:21.507) and demonstrated progressive improvement in neurological function. Post-rehabilitation, the patient achieved full behavioral responsiveness, including communication and self-care abilities. Notably, a marked reduction in negative SE values was observed in both the 1–20 Hz band (baseline: −1.438; 3-month follow-up: −1.029) and the 1–40 Hz band (baseline: −1.431; 3-month follow-up: −1.158), as illustrated in Figure 4.

### 3.6. Individual Electrophysiological-Metabolic Coupling Analysis

**Figure 5:**
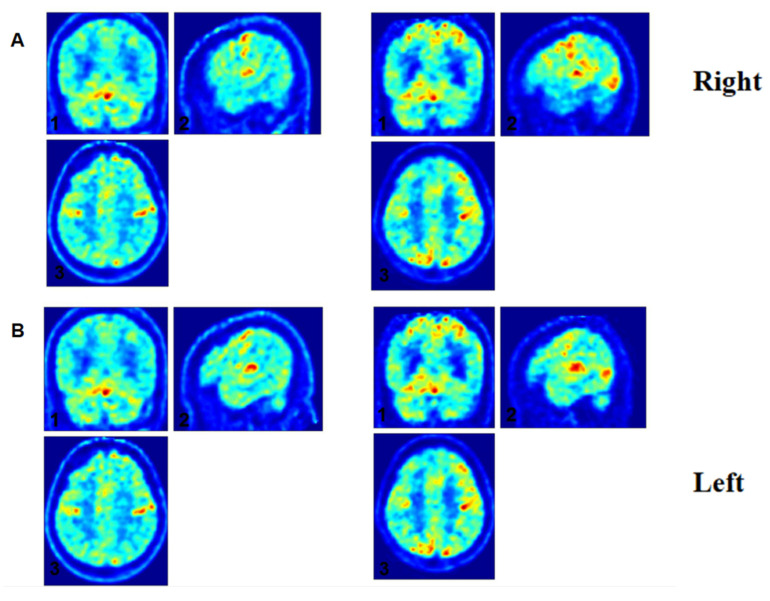
Longitudinal metabolic changes in parietal cortex of DoC Patient 13. Notes: Serial FDG-PET images demonstrate progressive metabolic reactivation in the inferior parietal lobule (IPL). (**A**) Right IPL: coronal (1), sagittal (2), and axial (3) views at baseline (left) and 3-month follow-up (right). (**B**) Left IPL: coronal (1), sagittal (2), and axial (3) views. Increased glucose metabolism (red = high metabolic activity) and expanded activated cortical areas are evident post-rehabilitation.

**Figure 6:**
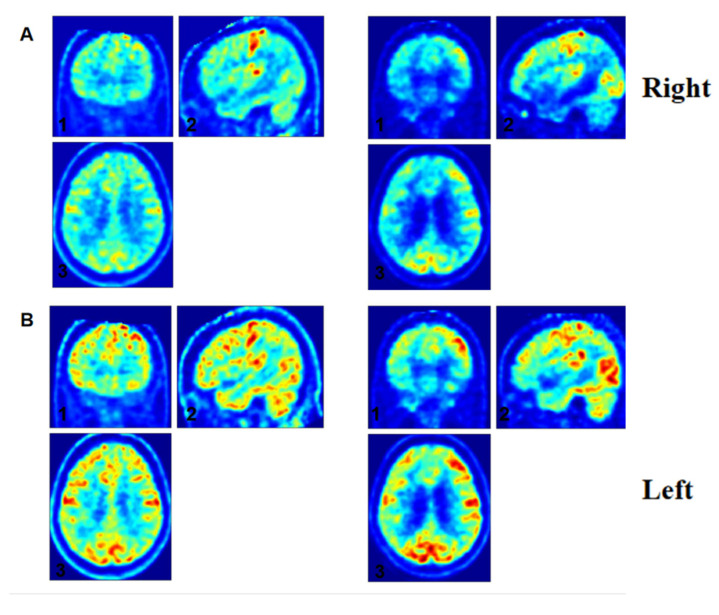
Longitudinal frontal metabolic changes in DoC Patient 13. Notes: Serial FDG-PET images demonstrate progressive metabolic reactivation in the dorsolateral prefrontal cortex (DLPFC). (**A**) Right DLPFC: coronal (1), sagittal (2), and axial (3) views at baseline (left) and follow-up (right). (**B**) Left DLPFC: coronal (1), sagittal (2), and axial (3) views. Increased glucose metabolism (red = high metabolic activity) and expanded activated cortical areas are evident post-rehabilitation.

Patient 13, a pDoC, exhibited paradoxical neurophysiological improvements despite minimal behavioral progress on the CRS-R. While CRS-R behavioral scores showed no significant improvement (baseline: 2-4-5-1-0-2, CRS-R index = 56.93667; post-rehabilitation: 2-4-5-2-0-2 with a 1-point increase in oromotor function, CRS-R index = 57.98667), multimodal neuroimaging revealed marked enhancements. Positron emission tomography (PET) demonstrated bilateral increases in metabolic intensity and spatial extent within frontal and parietal cortices (Figure 5 and Figure 6). Concurrently, SE values improved from −1.7561 to −1.6831, reflecting reduced 1/f slope steepness. Clinically, this neurophysiological-metabolic dissociation correlated with the emergence of active voluntary movements, suggesting preserved subclinical recovery undetected by behavioral assessments alone.

## 4. Discussion

This study systematically evaluates the diagnostic utility of spectral exponent (SE) in patients with pDoC, yielding three principal findings: (1) Narrowband SE (1–20 Hz) demonstrates superior discriminative validity across diagnostic groups (DoC vs. Controls) and subtyping precision (MCS vs. VS/UWS); (2) SE exhibits robust associations with both composite CRS-R indices and modality-specific visual subscale scores; and (3) SE may be a potential biomarker of consciousness that reflects consciousness level rather than the degree of structural brain damage solely. These findings collectively position SE as a promising electrophysiological biomarker for consciousness quantification, addressing critical limitations of behavior-dependent clinical assessments.

### 4.1. Neural Mechanisms and Frequency-Dependent Dynamics

The steepened SE profiles (more negative values) observed in DoC patients mechanistically bridge two foundational theories of consciousness: IIT and the Global Neuronal Workspace Theory (GNWT). According to IIT, consciousness arises from the thalamocortical system’s capacity for information integration [27], a process directly compromised of the shift in E–I balance toward inhibitory dominance. Computational models demonstrate that prolonged inhibitory postsynaptic potentials enhance low-frequency synchronization and accelerate PSD decay [22,23], a mechanism reflected in SE steepening. Crucially, the absence of SE differences between BI and HC cohorts (*p* > 0.999) underscores SE’s specificity to functional network dynamics rather than static structural damage. This aligns with GNWT’s emphasis on dynamic coordination of global networks as the neural correlation of consciousness [28]: Inhibitory dominance not only reduces thalamocortical information integration (IIT’s core postulate) but also disrupts large-scale frontoparietal coordination essential for GNWT’s neuronal workspace. Thus, SE uniquely quantifies consciousness through dual lenses—informational (IIT’s integration capacity) and dynamical (GNWT’s network coordination)—providing a unified biomarker for impaired consciousness.

Notably, SE steepening mirror GABAergic modulation patterns observed during propofol anesthesia [22,29], where subcortical PSD alterations temporally align with consciousness loss. This pharmacological parallelism suggests SE may serve as a surrogate marker of global inhibitory tone, though its exact relationship to neurotransmitter dynamics warrants further pharmaco-EEG investigations.

The diagnostic superiority of narrowband (1–20 Hz) SE arises from two neurophysiological constraints: (1) Dendritic Filtering Artifacts: Cortical pyramidal neurons exhibit low-pass filtering properties due to dendritic cable characteristics, inducing a spectral “knee point” (~20 Hz) that distorts broadband 1/f slope estimation [24,30]. Narrowband analysis minimizes contamination from attenuated high-frequency (>20 Hz) signals and (2) Thalamocortical Noise Confounders: Type C DoC patients often retain thalamic burst-suppression patterns that generate high-frequency (>20 Hz) spectral peaks, confounding broadband SE interpretation [15]. This observation parallels anesthesia studies where 20–40 Hz SE alterations reflect drug-specific GABA_A receptor effects rather than consciousness per se [19,31].

### 4.2. Behavioral Correlates and Alpha Oscillation Mediation

The robust SE-CRS-R correlation (r = 0.590, *p* = 0.021) extends Colombo’s work [19] by establishing SE as a complementary metric to PCI for consciousness stratification. Strikingly, the SE-visual subscale association (r = 0.684, *p* = 0.005) implicates alpha oscillations as dual mediators. For spectral slope contribution, alpha power reductions amplify SE negativity through their role as primary carriers of aperiodic components [32]. But for perceptual gating function, parieto-occipital alpha synchronization facilitates conscious visual perception via attentional modulation [33,34]; its disruption in DoC may degrade both visual awareness and SE profiles. This dual mechanism aligns with the “dynamic signature of consciousness” framework [35], wherein conscious states require precise spatiotemporal coordination of oscillatory processes—a coordination disrupted in SE-steepened DoC patients.

### 4.3. Prognostic Potential and Neuro-Behavioral Dissociation

Longitudinal tracking in Patient 5 (individual analysis) revealed a reduction in SE negativity (i.e., flattening of the 1/f slope) paralleling behavioral recovery, suggesting its utility as a dynamic prognostic biomarker. Intriguingly, Patient 13 exhibited neurophysiological-metabolic recovery (PET-confirmed prefrontal-parietal reactivation) despite stagnant CRS-R scores, highlighting SE’s capacity to detect covert neuroplasticity preceding behavioral recovery. This neuro-behavioral dissociation mirrors the “cognitive-motor dissociation” [4], positioning SE as a tool to identify candidates for thalamic deep brain stimulation or frontoparietal tDCS—interventions shown to enhance consciousness in structurally compromised but functionally preserved networks. Then, integrated SE-PET protocols could, thus, personalize neuromodulation targets.

### 4.4. Limitations

This study has limitations: (1) Sample size constraints: The relatively small cohort size (n = 15 DoC patients) reduces statistical power for subgroup analyses, particularly given the etiological heterogeneity of brain injuries (traumatic vs. non-traumatic). This limitation may obscure subtle differences in SE dynamics across injury subtypes or recovery trajectories. (2) Lack of multimodal validation: While SE demonstrated robust correlations with behavioral metrics (CRS-R), its neurobiological specificity remains partially inferred. The absence of concurrent fMRI or FDG-PET data limits our ability to directly link SE steepening to functional network disintegration or metabolic deficits in thalamocortical circuits. (3) Single-center retrospective design: The study’s retrospective, single-center design introduces potential biases in patient selection and protocol standardization. Prospective multicenter trials are needed to validate SE’s generalizability across diverse clinical settings and scanner hardware.

## 5. Conclusions

This study establishes SE as a global correlate of consciousness in DoC, demonstrating its capacity to distinguish E-I ratio differences between VS/UWS and MCS at the group level. These findings position SE as a promising biomarker for consciousness quantification. By capturing whole-brain spectral characteristics of neural noise, SE provides an objective diagnostic metric transcending behavioral subjectivity. Its strong visual subscale association further suggests potential as a specific biomarker for residual awareness assessment. Subsequent research should validate SE’s etiological specificity in larger cohorts and explore its integration with multimodal metabolic-electrophysiological markers.

## Figures and Tables

**Table 1:** Demographic and clinical characteristics of DoC patients.

Patient ID	Age	Sex	Etiology	Time Since Injury (Days)	CRS-R Subscale Scores	CRS-R Index	Diagnosis	ABCD EEG Typology
1	69	M	NTBI	490	0-2-2-1-0-2	13.17667	MCS	B
2	55	M	NTBI	174	1-0-2-1-0-1	4.50333	VS	B
3	51	M	TBI	419	2-4-5-2-0-2	57.98667	MCS	B
4	34	M	NTBI	62	1-0-2-1-0-2	4.83667	VS	B
5	16	M	NTBI	41	1-3-1-1-0-2	21.50667	MCS	B
6	49	M	TBI	419	1-3-2-1-0-2	22.54667	MCS	B
7	60	M	NTBI	214	1-3-2-1-0-1	22.21333	MCS	C
8	36	M	NTBI	171	1-0-2-1-0-1	4.50333	VS	B
9	72	M	TBI	307	1-1-5-1-0-2	30.88667	MCS	B
10	50	F	TBI	218	1-3-2-1-0-1	22.21333	MCS	C
11	53	F	NTBI	40	0-3-2-0-0-2	20.46667	MCS	B
12	54	M	NTBI	95	0-0-2-1-0-2	3.79667	VS	A
13	65	M	TBI	95	1-1-2-1-0-1	5.54333	VS	A
14	71	M	NTBI	102	0-1-2-1-0-2	4.83667	VS	A
15	65	F	TBI	476	1-2-1-1-0-2	13.17667	MCS	B

**Table 2:** Demographic and clinical characteristics of BI Controls.

Patient ID	Age	Sex	Etiology	Lesion	Injury Type (Cortical/Subcortical/Combined)
1	41	M	TBI	Right frontal lobe and left basal ganglia hematoma	Combined
2	37	F	TBI	Subarachnoid hemorrhage, left hemispheric cerebral hematoma	Combined
3	50	M	NTBI	Right frontal lobe hemorrhage	Cortical
4	42	M	NTBI	Right basal ganglia hemorrhage	Subcortical
5	58	M	NTBI	Left frontal lobe hemorrhage	Cortical
6	16	M	TBI	Left cerebellar hemisphere abnormalities and basilar artery stenosis	Combined
7	70	M	NTBI	Subarachnoid hemorrhage	Subcortical
8	49	M	NTBI	Left basal ganglia hemorrhage	Subcortical
9	60	M	TBI	Bilateral frontal lobe hematomas and subarachnoid hemorrhage	Combined

**Table 3:** Demographic and clinical characteristics across groups.

Characteristic		HC Group (N = 23)	BI Group (N = 9)	DoC Group (N = 15)	Statistical Analysis
Age (years) mean ± std		51.2 ± 12.2	47.0 ± 15.6	53.3 ± 15.4	F(2) = 0.580 *p* = 0.564
Sex (%)	Male	6 (26.1%)	8 (88.9%)	12 (80.0%)	χ^2^(2) = 15.752 *p* < 0.001 ***
	Female	17 (73.9%)	1 (11.1%)	3 (20.0%)	
Etiology (%)	TBI		4 (44.4%)	6 (40%)	χ^2^(1) = 0.667 *p* = 0.541
	NTBI		5 (55.6%)	9 (60%)	
Time since injury (days) mean ± SD			109.33 ± 79.07	221.53 ± 161.31	U = 37.00 *p* = 0.069

Abbreviations: TBI = Traumatic Brain Injuries; NTBI = Non-Traumatic Brain Injuries. SD = Standard Deviation. *** *p* < 0.001. Note: One-way ANOVA for age analysis; chi-squared for sex and etiology analysis; Mann–Whitney U test for time since injury analysis.

**Table 4:** Intergroup analysis of SE across frequency bands.

Frequency	HC Group	BI Group	DoC Group	H	*p*	Post Hoc Comparisons (Bonferroni-Adjusted)	BF_10_	η²
β_1–40 Hz_	−1.038 (0.109)	−1.129 (0.131)	−2.064 (0.601)	32.097	1.072 × 10^−7^	HC > DoC (*p* < 0.001) ***, BI > DoC (*p* = 0.0059) *	5.632 × 10^13^	0.81
β_1–20 Hz_	−1.063 (0.266)	−1.063 (0.246)	−2.028 (0.604)	29.738	3.487 × 10^−7^	HC > DoC (*p* < 0.001) ***, BI > DoC (*p* = 0.0006) ***	5.996 × 10^9^	0.71
β_20–40 Hz_	−2.118 (1.026)	−1.435 (1.203)	−1.643 (0.982)	7.579	0.023	DoC < HC (*p* = 0.0212) *	2.834	0.15

Note: Significance levels: *** *p* < 0.001, * *p* < 0.05

**Table 5:** Intragroup analysis of SE across broad- and narrow-band in DoC subgroups.

	MCS	VS/UWS	t	*p*	BF_10_	Cohen’s d
β_1–40 Hz_	−1.854 ± 0.271	−2.321 ± 0.331	−3.000	0.0102	5.096	1.580
β_1–20 Hz_	−1.791 ± 0.238	−2.445 ± 0.395	−4.025	0.0014	21.612	2.121
β_20–40 Hz_	−1.832 ± 0.545	−0.906 ± 0.665	2.961	0.0110	4.839	−1.561

**Table 6:** Correlation analysis between SE and behavioral scores in DoC patients.

Parameter	CRS-R Index		Visual Subscale	
	r	*p*	r	*p*
β_1–40 Hz_	0.569 *	0.027	0.566 *	0.027
β_1–20 Hz_	0.590 *	0.021	0.684 **	0.004
β_20–40 Hz_	−0.459	0.085	−0.631 *	0.012

Note: Significance levels: ** *p* < 0.01,* *p* < 0.05

## Data Availability

The data presented in this study are available on request from the corresponding author due to privacy.
